# Flow cytometry‐based characterization of underlying clonal B and plasma cells in patients with light chain amyloidosis

**DOI:** 10.1002/cam4.725

**Published:** 2016-04-25

**Authors:** Katharina Lisenko, Stefan O. Schönland, Anna Jauch, Mindaugas Andrulis, Christoph Röcken, Anthony D. Ho, Hartmut Goldschmidt, Ute Hegenbart, Michael Hundemer

**Affiliations:** ^1^ Department of Internal Medicine VUniversity of HeidelbergHeidelbergGermany; ^2^ Institute of Human GeneticsUniversity of HeidelbergHeidelbergGermany; ^3^ Department of PathologyUniversity of HeidelbergHeidelbergGermany; ^4^ Department of PathologyUniversity Medical Center Schleswig‐HolsteinKielGermany; ^5^ National Center for Tumor DiseasesUniversity of HeidelbergHeidelbergGermany

**Keywords:** AL‐amyloidosis, immunotherapeutical targets, lymphatic neoplasia, multi‐parameter flow cytometry

## Abstract

Systemic amyloid light chain (AL) amyloidosis is a life‐threatening protein deposition disorder; however, effective therapy can dramatically improve the prognosis of AL patients. Therefore, accurate diagnosis of the underlying hematologic disease is important. Multi‐parameter flow cytometry (MFC) is a reliable method to analyze lymphatic neoplasias and to detect even a small lymphatic clone. We analyzed the presence of clonal plasma cell (PC) and B cells in the bone marrow of 63 patients with newly diagnosed AL amyloidosis by MFC. We compared the results with the levels of monoclonal protein, the histopathology and cytogenetic results. As reference of light chain restriction, we used the immunohistochemical results of *κ* or *λ* positive amyloid deposits in various tissues. MFC identified underlying clonal lymphatic cells in all but two patients (61 of 63, 97%). Sixty‐one patients harbored malignant PCs, whereas B‐cell lymphomas were identified in two patients. Furthermore, MFC indicated at least one putative immunotherapeutical target (CD20, CD38, CD52, or SLAMF7) on malignant PCs in all but one patient. These results demonstrate that MFC is a reliable tool for an accurate diagnosis of the underlying hematologic disease and the detection of potential immunotherapeutical targets in patients with AL amyloidosis.

## Introduction

Amyloid light chain (AL) amyloidosis is a rare and life‐threatening protein deposition disorder and the most common type of systemic amyloidosis 1. It is characterized by the accumulation of monoclonal light chain in various organs as amyloid fibrils, predominantly in the heart, kidney, peripheral nerves, liver and gut 2, 3. The persistent accumulation of amyloidogenic *κ* or *λ* light chains, which are typically produced by a plasma cell (PC) clone, ultimately leads to severe organ damage and shortens survival 4, 5. In most of the cases, a small PC clone accumulates in the bone marrow (BM) with a median PC percentage of less than 10% 6, 7. However, in about 3% of AL amyloidosis patients, the disease occurs in the setting of a low‐grade B‐cell non‐Hodgkin lymphoma (NHL) 8. Cases of systemic amyloid deposition associated with lymphoplasmacytic lymphoma, chronic lymphocytic leukemia and marginal zone lymphoma have also been reported in the literature 9, 10, 11, 12.

The goal of therapy is to target the amyloidogenic PC or B‐cell clone and to halt the uncontrolled release of free light chains (FLCs), which can lead to improvement of organ function and survival. Given the different underlying diseases and therapeutic options in systemic AL amyloidosis, the identification of only the paraprotein in the serum and urine is insufficient. The amyloidogenic clone must also be detected and characterized for optimal therapeutic strategy 13. Due to the small size of the malignant clone, the diagnostic methods must be sensitive and capable of differentiating between clonal B cells and PC. Additionally, in terms of targeted therapy, diagnostic methods should also provide the possibility of target identification on the surface of the malignant cells.

Multicolor flow cytometry (MFC) is a widely available diagnostic technique that allows the differentiation of PC and B‐cell disorders while simultaneously detecting a broad array of surface and intracellular antigens at a high level of sensitivity. Compared with histopathological evaluation, BM aspirates are easier to obtain than biopsies, and MFC analysis is significantly faster than immunohistochemistry (IHC) techniques.

We retrospectively analyzed 63 consecutive patients with AL amyloidosis who were referred to our Amyloidosis Center from March 2014 to May 2015 and were thoroughly assessed using IHC for the amyloid deposits in organs, serum as well as urine immunofixation (IF), serum FLC test, BM cytology, BM MFC, BM IHC, and interphase fluorescence in situ hybridization (iFISH) on sorted CD138^+^ BM PCs. This study aimed to demonstrate the utility and sensitivity of MFC in the identification and characterization of underlying clonal cells in AL amyloidosis and, in particular, to evaluate the accuracy of this method in the detection of light chain restriction on a retrospective cohort of well characterized patients with AL amyloidosis.

## Methods

We retrospectively analyzed 63 patients who were newly diagnosed with systemic AL amyloidosis from May 2014 to May 2015 at our Amyloidosis Center at the University Hospital in Heidelberg, Germany. BM smears for cytomorphological quantification of PCs, IHC of BM biopsies and serum and urine IF measurements were performed as a part of the clinical routine. All patients gave written informed consent for the study. Approval was obtained by the Ethics Committee of the University Heidelberg.

### Histology and immunohistochemistry

The diagnosis of AL amyloidosis was confirmed histologically in every patient. Amyloid was detected by Congo red‐staining viewed under polarized light showing green birefringence and Congo red fluorescence. Immunohistochemical classification of amyloid was carried out as described elsewhere with commercially available monoclonal antibodies directed against AA amyloid and polyclonal antibodies directed against amyloid P‐component, *λ*‐light chain, *κ*‐light chain, fibrinogen, Aβ amyloid (all from DAKO, Hamburg, Germany) 14. In addition, we used non‐commercially available polyclonal antibodies directed against apolipoprotein AI (anti‐apoAI), transthyretin (ATTR3), *λ*‐light chain (AL1, AL3, and AL7), and *κ*‐light chain (AK3) 14. Immunohistochemical classification was carried out in blinded fashion, that is, without knowledge of the results of immunophenotyping testing.

### Immunophenotyping

For immunophenotyping, fresh BM aspirates were obtained as a part of the clinical routine. The BM aspirates were separated into 100 *μ*L aliquots. To detect malignant B cells, immunophenotyping was conducted with antibodies against k light chain (PE, R0436, Dako Cytomation), *λ* light chain (FITC, F0435, Dako Cytomation), CD19 (PE‐Cy7, HIB19, BD), and CD45 (APC‐Cy7, J33, BD). For PC immunophenotyping, an eight‐parameter flow cytometry analysis was performed using cytoplasmic anti‐*κ*, cytoplasmic anti‐*λ*, CD19, CD20, CD22, CD27, CD30, CD38, CD45, CD52, CD56, CD81, and SLAMF7 antibodies (Table 1). Fixation and permeabilization (FIX&PERM) Solutions A and B (Biozol, Eching, Germany) were used for cytoplasmic anti‐*κ* and anti‐*λ* staining, and BD FACS^™^ Lysing Solution was used for red cell lysis (BD, Heidelberg, Germany). Before measurement, the cells were washed twice and resuspended in 500 *μ*L of phosphate‐buffered saline (Life Technologies, Carlsbad, California). The measurement was performed on a FACSCanto^™^ II cell analyzer (BD, Heidelberg, Germany). The compensation matrix was calculated using BD CompBead particles (BD, Heidelberg, Germany), and the compensation setup tool in BD FACSDiva^™^ software was used. Data were analyzed using the Infinicyt^™^ software (Cytognos, Salamanca, Spain).

**Table 1:** Flow cytometry panel and antibody characteristics

Fluorochrome^a^								
Tube	Pacific blue	Pacific orange	FITC	PE	PerCP‐Cy5.5	PE‐Cy7	APC	APC‐H7
1	CD45 (2D15, BD)	CD138 (B‐A38, Biozol)	CD38 (HB7, BD)	CD56 (NCAM16.2, BD)	ß2‐micro‐ globulin (TU99, BD)	CD19 (HIB19, BD)	anti‐*κ* (TB28‐2, BD)	anti‐*λ* (1–155–2, BD)
2	CD45 (2D15, BD)	CD138 (B‐A38, Biozol)	CD38 (HB7, BD)	CD22 (SJ10, Beckman Coulter)	CD27 (L12828, BD)	CD19 (HIB19, BD)	CD30 (BerH8, BD)	CD81 (JS‐81, BD)
3	CD45 (2D15, BD)	CD138 (B‐A38, Biozol)	CD38 (HB7, BD)	CD52 (CF1D12, Life Technologies)	CD20 (L27, BD)	CD19 (HIB19, BD)	SLAMF7 (235614, R&D Systems)	CD81 (JS‐81, BD)

a Clone and manufacturer are indicated within parenthesis.

Aberrant B cells were identified based on the expression of CD19 and intracellular light chain restriction. PCs were identified based on the coexpression of CD38 and CD138 antigens. Malignant and normal PCs were differentiated based on intracellular light chain restriction and an analysis of aberrant CD45, CD19, and CD56 expression. When an intracellular light chain restriction was not evident, a back‐gating strategy of CD45− and CD19‐negative as well as CD56‐positive PCs was performed. Overall, positivity was defined when the antigen was expressed on ≥20% of analyzed PCs.

### Free light chain test

The concentrations of serum FLCs were measured by a latex‐enhanced immunoassay (Freelite TM Human Kappa Free Kit, The Binding Site GmbH, Schwetzingen, Germany) on a Behring 2 nephelometer as described elsewhere 15. The reference ranges were used according to the manufacturer's instructions as follows: 3.3–19.4 mg/L for *κ* light chain, 5.7–26.3 mg/L for *λ* light chain, and 0.3–1.6 for *κ*/*λ* ratio 16. The FLC test was considered positive when the criteria of both an abnormal FLC ratio and an elevation of the involved light chain above the respective upper range were met.

### Molecular cytogenetic testing

Molecular cytogenetic testing was performed as described previously 17. Briefly, CD138^+^ BM PCs were purified by auto‐magnetic‐activated cell sorting with anti‐CD138 immunobeads. For iFISH analyses, a panel of two‐color probe sets for the detection of numerical changes for the chromosome loci 1q21/13q14, 5p15/5q35, 8p21/19q13, 9q34/15q22, and 11q22.3/17p13, the IgH‐translocations t(11;14)(q13;q32), t(4;14)(p16;q32), t(14;16)(q32;q23) as well as an IgH‐breakapart probe were used. After hybridization according to the manufacturer's instructions (Kreatech, Amsterdam, Netherlands and MetaSystems, Altlussheim, Germany), a minimum of 100 interphase nuclei per probe was evaluated using an automated iFISH spot counting system (Applied Spectral Imaging, Edingen‐Neckarhausen, Germany). Hybridization efficiency was validated on interphase nuclei obtained from BM of a healthy donor and the thresholds for gains, deletions, and translocations were set at 10%. Hyperdiploidy was defined according to the criteria described by Wuilleme et al., which require trisomies of at least two of the three chromosomes 5, 9, and 15 18. Translocations t(4;14) and t(14;16) as well as del17p13 were defined as high‐risk aberrations 19.

### Statistical analysis

The results are presented as median, maxima, and minima. Data were compared using student's *t*‐test and the chi‐squared test. A *P*‐value ≤0.05 was considered statistically significant.

## Results

### Patient characteristics

We included 63 patients (37 male and 26 female) with newly diagnosed systemic AL amyloidosis. The median age was 62 (range 40–77) years. The demographic and disease characteristics are summarized in Table 2.

**Table 2:** Clinical characteristics of amyloid light chain (AL) amyloidosis patients

*n*	63
Age median, range (years)	62, 40–77
Gender	
Male	37 (59%)
Female	26 (41%)
Involved organs	
1–2	40
3–4	20
>4	3
Heart	43
Kidney	32
Liver	6
Spleen	1
GI tract	21
Soft tissue	30
Nervous system	14
Underlying disease	
Monoclonal gammopathy	19 (30%)
Smoldering multiple myeloma	34 (54%)
Symptomatic multiple myeloma	8 (13%)
Low grade B‐NHL	1 (1.5%)
Waldenstrom macroglobulinemia	1 (1.5%)
Heavy chain type	
IgG	17
IgM	4
IgA	9
IgD	1
none	31
n.a.	1
Light chain type	
*κ*	16
*λ*	47
Serum FLC median, range (mg/L)	
*κ* AL amyloidosis	
*κ* FLC	329, 24–9600
*λ* FLC	11, 0.4–35
*λ* AL amyloidosis	
*κ* FLC	12, 1–68
*λ* FLC	246, 32–3440
BM PCs median, range (%)*	
Cytology	10, 2–68
MFC	4, 0.2–34
Histology (*n* = 42)	n_<10% PCs_ = 23
	n_≥10% PCs_ = 19

The table presents age, gender, number of involved organs, underlying disease, heavy and light chain type, serum FLC, and the number of PCs in BM aspirates in AL amyloidosis patients with * monoclonal gammopathy (MG) and multiple myeloma (MM) as underlying disease. The heavy chain type was determined by serum immunofixation. BM, bone marrow; B‐NHL, B non‐Hodgkin lymphoma; FLC, free light chain; Ig, immunoglobulin; MFC, multicolor flow cytometry; PC, plasma cell.

### Detection of amyloidogenic clones in patients with systemic AL amyloidosis

To detect the amyloidogenic clone, we performed an MFC analysis of the BM aspirates of all patients (*n* = 63). We identified an aberrant PC population in 61 and a light chain‐restricted B‐cell population in two of the 63 patients.

**Figure 1:**
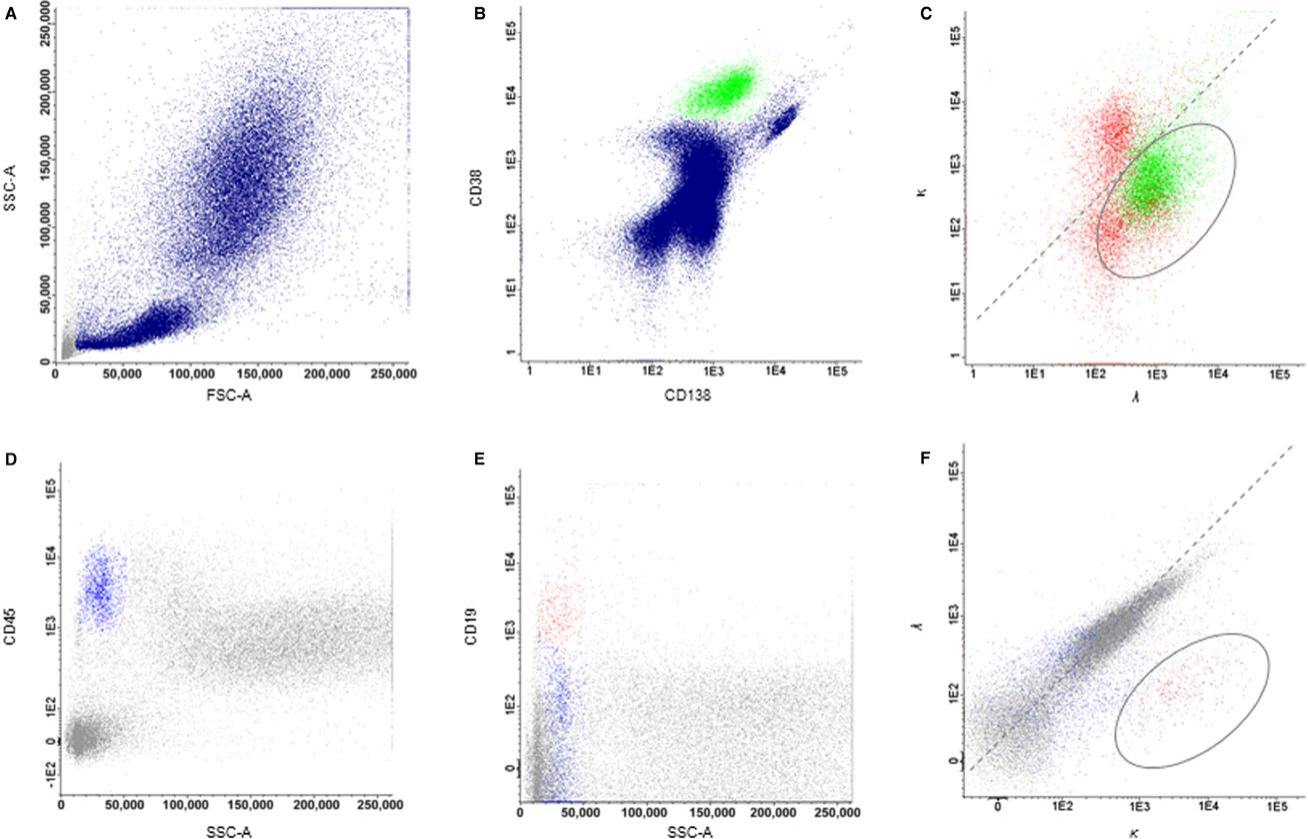
Identification of amyloidogenic clone by Multi‐parameter flow cytometry. Gating strategy for the identification of light chain‐restricted plasma cells (PCs) (A–C) and B cells (D–F). PCs (green, B) were identified as CD38− and CD138‐positive cells among leukocytes (blue, A). PCs were anticipated as monoclonal when light chain restriction was observed. Figure C depicts a *λ* light chain‐restricted PC population (green), whereas *κ* and *λ* expression was noted on polyclonal B cells (red). B cells (green, E) were detected as a CD19‐positive population among leukocytes (blue, D). Light chain restriction on B cells was identified with the *κ*/*λ* gate (F). CD, cluster of differentiation; FSC‐A, forward scatter area; SSC‐A, side scatter area.

**Figure 2:**
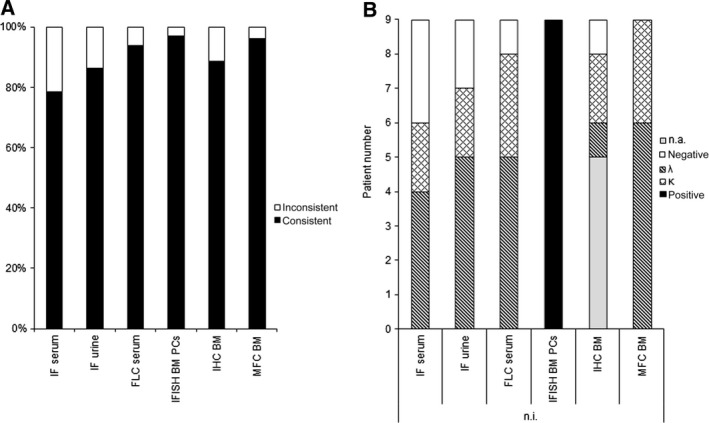
Accuracy of light chain detection by various methods in patients with amyloid light chain (AL) amyloidosis and underlying plasma cell (PC) disorder. Consistent and inconsistent light chain recognition by immunofixation, serum free light chain test, iFISH of bone marrow (BM) PCs, BM immunohistochemistry (IHC) and BM Multi‐parameter flow cytometry compared with light chain restriction detected by IHC of amyloid deposits in *κ* and *λ* AL amyloidosis patients (*n* = 52, A). Light chain or clonality detection in AL amyloidosis cases without IHC identification of *κ* or *λ* amyloid deposits based on various methods (*n* = 9, B).

To evaluate the accuracy of MFC in detecting PC light chain restriction, we compared the MFC results with serum and urine IF, the serum FLC, and the IHC of BM biopsies (Table 3). We referred to the light chain type identified within amyloid deposits in involved organs by IHC as a reference for light chain restriction. We first evaluated all *κ* and *λ* AL amyloidosis cases in which IHC of amyloid deposits was available (*n*_*κ*_ **= 12, *n*_*λ*_ **= 40). In these cases, serum and urine IF identified a consistent light chain restriction in 78% (40/51) and 86% (44/51) of samples, respectively. The serum FLC test was positive in all *κ* (100%, 12/12) and all but four *λ* AL (90%, 36/40) amyloidosis cases. MFC detected the amyloidogenic clone in all *κ* (100%, 12/12) and in 95% (38/40) of patients with *λ* AL amyloidosis (Fig. 1A–C). We were unable to demonstrate a light chain restriction by MFC in two patients with *λ* AL amyloidosis, although we detected a relevant aberrant CD45low, CD19low PC population. BM IHC detected a clonal disease with the same light chain restriction as IHC of amyloid deposits in 89% (31/35) of the examined cases. Figure 2A presents the percentage of consistent and inconsistent results obtained by the different methods.

**Table 3:** Clonality detection by IF, FLC, iFISH, IHC, and MFC in patients with amyloid light chain (AL) amyloidosis and underlying PC disorder

IHC organ biopsy	IF serum	IF urine	FLC serum	iFISH BM PCs	IHC BM	MFC BM
*κ*,* n* = 12						
*κ*	7	9	12	–	8	12
Positive	–	–	–	11	–	–
Negative	2	2	0	1	1	0
n.o.s.	2	1	0	0	0	0
n.a.	1	0	0	0	3	0
*λ*,* n* = 40						
*λ*	33	35	36	–	23	38
Positive	–	–	–	37	–	–
Negative	6	4	4	2	3	2
n.o.s.	1	0	0	0	0	0
n.a.	0	1	0	1	14	0
n.i., *n* = 9						
*κ*	2	2	3	–	2	3
*λ*	4	5	5	–	1	6
Positive	–	–	–	9	–	–
Negative	3	2	1	0	1	0
n.o.s.	0	0	0	0	0	0
n.a.	0	0	0	0	5	0

We analyzed clonality in 61 patients with AL amyloidosis and underlying PC disorder. The light chain type of the amyloid was identified by organ biopsy IHC in 52 patients (12 *κ* and 40 *λ*). In nine cases, IHC of organ biopsy was not able to classify amyloid deposits or was not available. Six different methods of clonality detection are compared. Number of cases with a positive, negative, not otherwise specifiable, or not available results are shown. BM, bone marrow; FLC, light chain; IF, immunofixation; iFISH, interphase fluorescence in situ hybridization; IHC, immunohistochemistry; MFC, multicolor flow cytometry; n.a., not available; n.i., not identified; n.o.s., not otherwise specified; PC, plasma cell.

In a second step, we focused on AL amyloidosis cases in which the amyloid type was not analyzed or identified by IHC (*n* = 9, Table 3, Fig. 2B). In three of these cases, IHC of amyloid deposits could not otherwise specify a light chain restriction, while in remaining six cases IHC of amyloid deposits was not available. A BM biopsy for IHC was not available in five patients and did not show a light chain restriction in one of these nine cases. In all patients BM MFC was able to identify a clonal light chain‐restricted PC population congruent with either serum FLC test and/or serum and urine IF.

In one case, MFC identified a *κ*‐restricted B‐cell population (Fig. 1D–F). This result was congruent with the detection of IgM *κ* in the serum IF and Bence Jones protein *κ* type, in the urine IF as well as a *κ* light chain positive serum. BM IHC did not exhibit evidence of light chain‐restricted B cells or PCs. In another patient, MFC identified a *λ*‐restricted B‐cell population. Further immunophenotypic and molecular characterization revealed a MYD88‐positive lymphoplasmacytic lymphoma. The detection of a *λ*‐restricted lymphocyte population by MFC in this patient was congruent with the detection of IgM *λ* in the serum IF and serum FLC test positive for *λ*. IHC of the soft tissue biopsy revealed *λ* amyloid deposits, while BM IHC was not performed.

### Chromosomal aberrations in bone marrow PCs as another marker of clonality in AL amyloidosis

For further characterization of the underlying clonal disease, a cytogenetic analysis of CD138^+^ BM PCs was performed. BM iFISH was available in all but one case with PC as underlying BM disorder (iFSH in *n* = 60). Translocation t(11;14) was the most frequent aberration and was found in 38 patients (63%). Deletion, del13q14 was present in 16 cases (27%). Multiple myeloma (MM) high‐risk aberrations were found in five patients (8%). Although translocation t(14;16) and del17p13 were identified in two cases, respectively, t(4;14) was found in only one case. Hyperdiploidy defined according to the criteria described by Wuilleme et al. was found in nine AL amyloidosis patients (15%). Other aberrations were present in 32 cases (53%). Overall, typical chromosomal aberrations of PC dyscrasias were found in 95% of AL amyloidosis patients (57/60) with a light chain‐restricted BM PC population identified by MFC as the amyloidogenic clone (Fig. 2, Table 3).

### Target identification on aberrant PCs in AL amyloidosis

**Figure 3:**
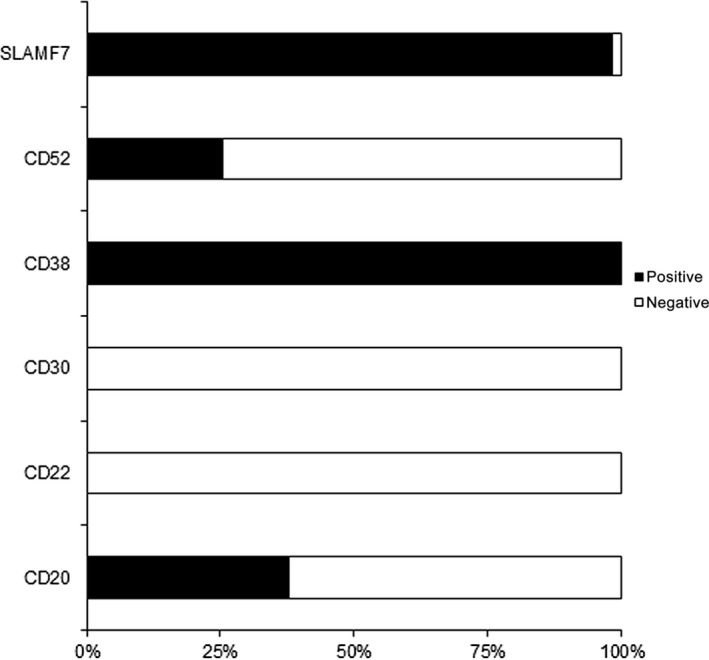
Surface targets in bone marrow plasma cells (PCs) in amyloid light chain (AL) amyloidosis. SLAMF7, CD52, CD30, CD22, and CD20 expression on PC in AL amyloidosis with a PC amyloidogenic clone (*n* = 61).

To characterize PC targets for potential treatment options in systemic AL amyloidosis with an aberrant PC population as the amyloidogenic clone, we evaluated the expression levels of SLAMF7, CD52, CD30, CD22, and CD20 on BM PCs by MFC in an increased number of patients (Fig. 3), 20. SLAMF7 was expressed in 98% of cases. CD52 was expressed in 25% of cases, and CD20 was expressed in 38% of cases. Because PCs were identified by CD138 and CD38 co‐expression, CD38 was identified in all cases. Neither CD22 nor CD30 was expressed in any of the samples. CD20 positivity significantly correlated with the presence of t(11;14) (*P* < 0.01): CD20 positivity was observed in 54% (20/37) of t(11;14) positive and only in 5% (1/20) of t(11;14) negative cases.

## Discussion

Systemic AL amyloidosis is a severe and life‐threatening disease that requires comprehensive and precise diagnostic evaluation 21. Amyloid deposits must be detected and typed, and the amyloidogenic clone should be identified to correctly diagnose the underlying hematologic disease and select appropriate and targeted treatment options. We demonstrate here in a large cohort that BM MFC can rapidly and sensitively identify either aberrant B cells or PCs as the amyloidogenic clone in AL amyloidosis patients, even when they are small.

BM MFC analysis identified an aberrant PC clone in 61 out of 63 examined (97%) AL amyloidosis patients. This result is in agreement with that of Paiva et al., who demonstrated the utility of MFC in AL amyloidosis patients with a malignant PC clone and identified aberrant PCs in 97% of patients with AL amyloidosis 22. Their results exceed the sensitivity of BM MFC analysis demonstrated by Hu et al. In an analysis of 51 AL amyloidosis cases, this group identified a light chain restriction in only 61% of cases 23. To date, the ideal method for the identification of monoclonal PCs in PC dyscrasias still lacks consensus, and different methods exist for the detection of aberrant PCs. Although Matsuda et al. demonstrated that monoclonal PCs in systemic AL amyloidosis can be identified by their aberrant phenotype (CD38+, CD56+, and CD19−) 24, we demonstrate for the first time that light chain restriction and back‐gating of CD45‐negative, CD19‐negative, and/or CD56‐positive PCs into the *κ*/*λ* gate successfully detects the aberrant PC clone. Specifically, the comparison between the light chain restriction identified on BM PCs by MFC and those identified by IHC of amyloid deposits exhibit a high level of congruence: BM MFC correctly identified the light chain restriction in 100% of *κ* and 95% of *λ* AL amyloidosis cases. Additionally, MFC from the BM was not only able to correctly identify the light chain restriction but also aided in the identification of amyloidogenic clone when the IHC and BM biopsies was either not otherwise specified or not available. It should be noted that some pathologists use FISH technique for the detection of monoclonal B cells, so although it is not recommended in the guidelines for the detection of MM cells 25, this analysis might have improved the specificity and sensitivity of IHC in this study. The light chain restriction identified on the PCs from the BM by MFC was always consistent with IHC, urine/serum IF, or serum FLC test results in AL amyloidosis cases in which the amyloid type was not identified by IHC. Moreover, by identifying even a very small amyloidogenic clone, BM MFC can also be useful for the AL amyloidosis patients presenting with an extremely low burden of PCs where the diagnosis sometimes is made based on an isolated positive IF, abnormal serum FLC ratio, or small clonal population of PC in the BM on IHC 26.

We used in this series also iFISH results as a marker for clonality. We could identify a typical PC disorder aberration in 95% of the patients, which confirmed our previous reports 17. Interestingly, our MFC sensitivity was in the same range and if taken together we could detect clonal PC in 100% of the cases.

Furthermore, we demonstrate that MFC provides useful information to specify immune‐chemotherapy in systemic AL amyloidosis. SLAMF7, CD20, and CD52 were expressed in 98%, 38%, and 25% of AL amyloidosis cases with light chain‐restricted PCs as the amyloidogenic clone, respectively. The high percentage of CD20‐positive cases is consistent with the immunophenotyping analysis performed by Deshmukh et al., who noted that CD20 was expressed in approximately 40% of the analyzed AL amyloidosis patients 27. CD20‐positive PCs in AL amyloidosis might represent a novel target for treatment with anti‐CD20 antibodies. Furthermore, alemtuzumab, an anti‐CD52 antibody 28, or elotuzumab, a humanized monoclonal antibody against the surface antigen SLAMF7 29, may offer new treatment options in systemic AL amyloidosis for positive patients. We observed a highly significant correlation between positivity for CD20 and t(11;14). Consistent with Garand et al. who showed that PCs in over 50% of t(11;14)‐positive MM cases exhibit a lymphoplasmacytoid morphology, our findings suggest that t(11;14) in AL amyloidosis might also be associated with a lymphoid PC phenotype 30.

In addition, it was possible to discriminate between malignant PC and B cells, so we identified two AL amyloidosis patients with a clonal B‐cell population in our study. This low frequency is consistent with the observation that AL amyloidosis rarely occurs (2–4%) in the setting of a NHL 31. The identification of a light chain‐restricted lymphocyte clone was congruent with the detection of monoclonal IgM *κ*/*λ* based on serum IF and positive FLC test in these patients and confirms the finding of an amyloidogenic B‐cell clone by MFC. Based on this finding, the two patients received lymphoma‐tailored therapy instead of a PC disorder‐adapted therapy.

Overall, we demonstrate the great practicability and applicability of BM MFC in patients with AL amyloidosis as a part of clinical routine to detect light chain‐restricted lymphatic clones. We also demonstrate that the MFC analysis of BM aspirates is a very sensitive method to distinguish B‐cell lymphomas from PC clones, making a decisive contribution to diagnosis. Regarding recent results about the necessity of minimal residual disease analysis in MM, MFC‐based analysis might also open the door in AL amyloidosis for detection of small malignant clones. In the long run, achieving a complete remission in AL amyloidosis to prevent the further production of amyloid might be even more important than in MM. In addition, MFC could detect a potential target for immunotherapeutic approaches on the surface of the malignant PC (SLAMF7, CD52, CD30, CD38, CD22, and CD20) in approximately all patients.

## Conflict of Interest

Katharina Lisenko: no conflict of interest. Stefan Schönland: received financial support for participation of congresses and research projects by Janssen and honoraria for talks by Janssen and Celgene. Anna Jauch: no conflict of interest. Mindaugas Andrulis: no conflict of interest. Christoph Röcken: no conflict of interest. Anthony D. Ho: Research funding from, and Membership on Advisory Board of Genzyme/Sanofi‐Aventis. Hartmut Goldschmidt: Research Support (Institution): Celgene, Janssen, Chugai, Novartis, BMS, Millennium; Advisory Boards (Institution): Celgene, Janssen, Novartis, Onyx, Amgen, Takeda, BMS; Honoraria: Celgene, Janssen, Novartis, Chugai, Onyx, Millennium. Ute Hegenbart: received financial support for participation of congresses by Janssen and honoraria for talks by Janssen and Celgene. Michael Hundemer: received financial support for participation of congresses by Baxalta, Celgene, and Amgen, financial support for research projects by Celgene, Chugai and Genzyme and honoraria for talks by Celgene.
